# The effect of static stretching exercises on hip range of motion, pain, and disability in patients with non-specific low back pain

**DOI:** 10.1186/s40634-021-00371-w

**Published:** 2021-07-27

**Authors:** Mohamadreza Hatefi, Farideh Babakhani, Mohadeseh Ashrafizadeh

**Affiliations:** 1 Department of Biomechanics and Sport Injuries, Faculty of Physical Education, Kharazmi University, Tehran, Iran; 2 Department of Sports Injury and Corrective Exercise, Faculty of Physical Education, Allameh Tabataba’i University, Tehran, Iran; 3 Department of Sports Injuries and Corrective Exercises, Faculty of Physical Education and Sports Sciences, University of Guilan, Rasht, Iran

**Keywords:** Oswestry disability questionnaire, Exercise therapy, Pain, Active stretching

## Abstract

**Purpose:**

The benefits of providing static stretching exercise targeting the hips in patients with non-specific Low Back Pain (NSLBP) are not well established. The objective of the study was to verify the effects of static stretching on function, pain and range of motion on patients with non-specific Low Back Pain (NSLBP).

**Methods:**

Thirty females with NSLBP were randomly assigned to two control (n = 15) and experimental (n = 15) groups. The experimental group received 3 stretch practice sessions per week for a period of 8 weeks. The Oswestry low back pain Disability Questionnaire (ODI), visual analog scale (VAS), and passive hip range of motion (PROM) were employed before and after the intervention.

**Results:**

The results of mixed model analysis of variance indicate that the group × time interactions was not significant (p > 0.05) for all measurement outcomes. However, there was a main effect for Time (ODI: p = 0.002, VAS: p = 0.001, PROM-R: p = 0.016, PROM-L: p = 0.001). Such that the ODI, VAS, PROM-R, and PROM-L were showed significant differences before and after the intervention in the experimental group.

**Conclusions:**

The results demonstrated a significant difference in PROM, pain, and disability after 8 weeks of stretching exercises in participants with NSLBP and limited hip extension. Therefore, it would be reasonable to infer that NSLBP might be partly related to hip flexors tightness.

## Introduction

Nonspecific low back pain (NSLBP) is a common condition, although the mechanisms of its occurrence are still not totally clear. Most people experience NSLBP more than once in their lifetime [1, 2]. Recently more attention has been paid to the hip and its potential contributions to NSLBP [3]. The emphasis in the past studies has mainly been placed on motor control, endurance, and strength factors in relation to NSLBP, and Limited focus is given on hip mobility and its potential contribution in patients with NSLBP [3].

Hip range of motion (ROM) is one of the vital movements for the natural load distribution mechanism in the hip and the function and loading of the spine and hip [2, 3]. Also, it is known that excessive anterior pelvic rotation occurs to compensate for lack of hip extension [4–6], and evidence suggests that a limited hip extension may alter the timing mechanism and motor activation of the lumbar spine [3, 7]. Further, a lack of hip extension may be associated with tightness in the hip flexor muscles [1, 2, 8, 9]. In this regards, tightness of hip flexor muscles has been recognized as a risk factor for various musculoskeletal injuries in the lower extremities [10–12]. Lastly, for patients with NSLBP that are sensitive to spinal extension, tightness of hip flexors may lead to performing spinal movements that bias increased spinal extension, as the patients lack movement options due to their hip extension limitations [9]. According to Winter et al. [11] and Roach et al. [3], hip passive range of motion (PROM) in people with NSLBP is on average 10° lower than that in healthy people. On this basis, static stretching for anterior hip muscles is occasionally chosen as a treatment option to improve the extensibility of these muscles and reduce excessive mechanical stress to the lumbar spine during particular movements of patients with NSLBP [5].

To date, there is no evidence regarding the effect of static stretching exercises for hip flexor muscles on hip ROM, pain, and disability of patients with NSLBP. In this regard, restricted hip flexor mobility has been clinically defined as the inability of the individual to achieve full hip extension during the modified Thomas test position [13]. In rehabilitation practice, stretching of hip flexor muscles has been acknowledged as effective in addressing limited hip extension ROM [11, 12, 14]. A variety of stretching techniques have been described in the literature [12, 14]. Among many methods of stretching, static stretching is presented as a safer and more effective method because it does not exceed the normal range of motion of joints. It does not require a high level of fitness, and causes less muscle pain [15]. Additionally, it has been demonstrated that a significant difference in hip extension exists in patients with NSLBP compared to controls group [3] and the strong correlation between limited hip extension and compensatory lumbar rotation suggests a risk of micro-trauma due to compensatory lumbar rotation [2].

Therefore, it may be important to consider hip mobility restrictions and their potential impact on pain and disability in patients with NSLBP. The purpose of the present study was to examine the effect of stretching exercises on hip passive ROM (PROM), Pain, and disability in patients with NSLBP. The authors hypothesized that significant differences in hip PROM, pain, and disability would result between pre and post stretching intervention protocols in the participants with NSLBP.

## Materials and methods

### Participants

According to G*Power software v 3.1 (Franz Faul University of Kiel, Germany), with a power of 0.8, effects size of 0.29 and an alpha level of 0.05, 26 females with NSLBP were required in this study. Accounting for a dropout rate/loss to follow up to 15%, a total of 30 female participants were required in this study. Afterward, the participants were randomly allocated to the experimental and control groups in a 1:1 ratio (Table 1); this was done via the computer-generated random allocation number by an investigator who was not involved in the study. Also, participants were invited to the present study through the board of *** University. Inclusion criteria were: tightness of hip flexor muscles (i.e. unilateral tightness of hip was excluded), experiencing chronic NSLBP for at least 3 months, pain radiating no further than the buttock, female between 25 and 40 years of age, and no experience with surgical treatments for disc herniation, spinal bifida, or spinal stenosis. Also, tightness of hip flexor muscles in the current study was identified as a participant demonstrating a bilateral hip extension angle between + 5° to + 15° above the Table surface during the modified Thomas test. Participants were excluded if they: diagnosis of systemic metabolic and/or neurological disorder, neuromuscular disorders, pregnancy, be in treatment for NSLBP in the same period of the interventions. Also, participants who were absent for more than three consecutive training sessions were excluded from the study.

**Table 1:** Participant Demographics

Characteristic	Group, Mean ± SD		*P* value
	Experimental (*n* = 15)	Control (*n* = 15)	
Age (Years)	26.27 ± 2.13	26.43 ± 2.57	0.18
Height (cm)	165.21 ± 5.18	164.65 ± 4.28	0.47
Body mass (kg)	74.51 ± 5.61	73.19 ± 4.81	0.84
BMI (kg/m2)	23.15 ± 3.15	23.32 ± 4.25	0.36

Prior to participants in the study, all participants were given an explanation of the study objectives and procedures, and provided written informed consent to participants. Ethical approval was granted by the ***University ethics boards in accordance with the Declaration of Helsinki.

### Procedures

In the present study, the experimental group was received the stretching interventions program and performed for eight weeks, three sessions a week on even days, while the control group did not receive the intervention. The stretching interventions program were performed for Approximately 20 min per session including: 1- Stretch in the modified Thomas test position, 2- Modified launch stretch, 3- Lifting the leg while lying in a prone position with the knee bent, 4- Lifting the leg in a prone position with a straight knee (Table 2).

**Table 2:** Stretching exercises program

1. Stretch in the modified Thomas test position The subject lies in the supine position on the table and took the modified Thomas test ' position. Meanwhile, the opposite leg was held actively by the participants with the hip and knee in a flexed position against the chest to stabilize the pelvis during the stretching exercise. First, the only force that leads to stretching flexor muscles hip was the weight of the tested limb and progressively a little force was applied in the direction of the knee extension to stretch. The 30-s stretch is performed with 8 s of rest and 10 repetitions
2. Modified launch stretch Like the half-kneeling position on the floor with a towel or pillow under the knee. The body is straight and by going forward and bending the hip, maximum tension is created in the hip on the side where the knee is bent. The 30-s stretch is performed with 8 s of rest and 10 repetitions [11]
3. Lifting the leg while lying in a prone position with the knee bent The subject lies in the prone position with the knee flexed to 90° to relax the hamstring, then contract the gluteal muscles as much as possible and lift the hip. The pillow is placed under the abdomen for 30 s with 8 s of rest and 10 repetitions [11]
4. Lifting the leg in a prone position with a straight knee Like the above exercise with the difference that the knee is straight. The subject lies in the prone position with the knee extended, then contract the gluteal muscles as much as possible and lift the hip. The pillow is placed under the abdomen for 30 s with 8 s of rest and 10 repetitions [11]

Written instructions were provided to each participant, describing the stretching techniques and the program details. After explaining the procedures, the stretch was demonstrated by the researcher. The participant was then asked to perform the stretch in the presence of the researcher to ensure the proper technique. Exercises started at a low level and gradually progressed, and the participant did the exercises according to their abilities. The intensity of exercises for each participant was controlled based on the exercise tolerance threshold and pain. By continuing the exercises, participant did the exercises with more repetition and decrease rest period without feeling pain or fatigue. This, on the one hand, motivated the participants and, on the other hand, maintained the principle of progressive resistance in the exercises. The progress of the exercises was at the same level for all participants and they were advised to do the exercises as long as they did not feel pain or discomfort. Whenever it was required, the selected exercises were modified for participants who felt pain during those exercises or were unable to maintain their proper posture.

The pre-and post-intervention measurements employed in the current research were: ODI, VAS, and hip PROM measurement. Both these measures were assessed at the start of the study and at the end of the 8 weeks of intervention. Also, the independent investigator was used to increase the feasibility of blinded assessment.

Oswestry low back pain Disability Questionnaire (ODI): The Disability index of NSLBP was evaluated using the ODI. The modified ODI is a 10 question condition-specific measurement of pain and disability for individuals with NSLBP. Each question is scored from 0 to 5 and summed for determination of total score, which is multiplied by 2 and expressed as a percentage [16].

VAS measurement: The pain level was evaluated using a visual analog scale (VAS; 0–10 cm). Zero indicated no pain and 10 cm indicate the worst pain level. The VAS score of 1.7 cm is reported as a minimal clinically important difference [17].

Hip PROM measurement: To measure the angle of the hip extension PROM, the universal goniometer was used while the participants were positioned supine and a modified Thomas test was performed. The modified Thomas test, typically used to measure the flexibility of the hip flexors to measure hip extension PROM, has been found to possess good reliability [18, 19]. The measured hip was positioned at the end of the table and the tested leg was then cantilevered over the edge of the table with the end feel resulting from the effects of gravity. The opposite leg was held actively by the participants with the hip and knee in a flexed position against the chest to stabilize the pelvis during the assessment. Instructions were provided for participants to pull their knee straight toward their head to avoid any abduction. In addition, participants were provided augmented feedback to maintain a neutral pelvis throughout the evaluation, which was accomplished with consistency in keeping knee firmly against the chest. To measure hip PROM, the goniometer axis is located on the outer surface of the hip joint, i.e. on the large femoral trochanter. The arm close to the trunk is placed along the outer midline of the pelvis and the arm far from the trunk is located along the outer midline of the hip with reference to the external epicondyle. Numbers were considered negative if the outer midline of the hip was higher than the horizon line, and positive numbers were considered if it was below the horizon line [3]. It is noteworthy that hip PROM was assessed by the same person for all participants.

### Statistical analysis

Descriptive analysis (mean and standard deviation) was performed on all the variables. The Shapiro–Wilk test was used to ascertain whether the data showed normal distribution. Therefore, a 2 × 2 mixed model analysis of variance was conducted. The between-subjects factor was group (control, experimental) and the within-subject factor was the time (pre-test, post-test). The Cohen d coefficient was used to determine effect size between groups. Effect size was interpreted as follows: d = 0.80 (large), d = 0.50 (medium), d = 0.20 (small). Statistical significance set a priori at p < 0.05. The Statistical Package for the Social Sciences (SPSS version 18.0, Microsoft Corp., Redmond, WA) was used for all the statistical analyses.

## Results

No significant differences were found for age, height, weight, and BMI between the groups (p > 0.05). The results of mixed model analysis of variance indicate that the group × time interactions and main effect for group were not significant (p > 0.05) for all measurement outcomes. However, there was a main effect for Time (ODI: p = 0.002, VAS: p = 0.001, PROM-R: p = 0.016, PROM-L: p = 0.001). Such that the post-intervention had more hip PROM in both legs (Right: -0.75 ± 0.96, Left: -1.23 ± 1.93) than the pre-intervention (Right: -5.41 ± 1.74, Left: -5.87 ± 1.76) in the experimental group. Also in this group, subjects exhibited less pain in the post-intervention (3.12 ± 1.28) than in the pre-intervention (4.70 ± 1.11) based on the VAS index, and exhibited less disability in the post-intervention (16.66 ± 5.86) than in the pre-intervention (29.51 ± 8.66) based on the ODI index. But no significant difference was found between before and after the intervention in the control group (Table 3).

**Table 3:** A comparison of pre- and post-intervention data in the two groups

Variable	Group						P-value		
	Control			Experimental					
	Mean ± SD		Effect Size	Mean ± SD		Effect Size	Time Main Effect	Group Main Effect	Group × Time Interaction
	Pre-test	Post-test		Pre-test	Post-test				
**ODI (%)**	28.01 ± 4.69	24.76 ± 4.93	0.54	29.51 ± 8.66	16.66 ± 5.86	0.68	0.002	0.451	0.568
**VAS (cm)**	5.11 ± 1.43	4.85 ± 1.08	0.61	4.70 ± 1.11	3.12 ± 1.28	0.61	0.001	0.148	0.918
**Hip PROM (Right) (°)**	-6.07 ± 1.97	-4.03 ± 1.21	0.49	-5.41 ± 1.74	-0.75 ± 0.96	.73	0.016	0.176	0.321
**Hip PROM (Left) (°)**	-5.01 ± 1.20	-4.26 ± 1.23	0.59	-5.87 ± 1.76	-1.23 ± 1.93	0.57	0.001	0.218	0.467

*p* ≤ 0.05, Oswestry Low Back Disability; ODI, visual analog scale; VAS, Passive Range of Motion; PROM

## Discussion

We investigated the effects of the stretching exercise program on ODI, VAS, and hip PROM in participants with NSLBP. The results of the study indicated the improved ODI, VAS, and PROM in participants who received stretching protocol. Previous studies have reported that change in the hip extension ROM have been associated with lower extremity injuries Including NSLBP [2]. In this regard, several studies have noted a relationship between NSLBP and anterior hip tightness. Roach et al. [19] noted on average a difference of 10° in hip extension ROM between those with NSLBP and controls, suggesting that the pain of this participants may be due to hip flexors tightness. Pattelma et al. [20] demonstrated that patients with both sub-acute LBP and NSLBP had significantly shortened hip flexors than those without NSLBP. Kim et al. [2] noted that the Strong correlations were observed among the degree of hip extension limitation, degree of hip asymmetry, pain intensity, ODI, and compensatory lumbar extension in participants with NSLBP. Also, the relationship between hip flexibility and NSLBP is important because the hip muscles act as an important link between the lower limbs and the trunk, and exert forces from the lower limbs to the spine as well as from the spine.

Therefore, it seems that improvement in extension flexibility of the hip joint can be considered as more efficient management of NSLBP. But a study was needed to clarify this issue. However, in a similar study, Mine et al. [5] examined the immediate effects of stretching for iliopsoas muscles on the limited hip extension in participants with NSLBP. They suggested that a single set of active static stretching might not immediately improve hip extension or reduce lumbopelvic movements of patients with NSLBP. The results of this study were not consistent with the present study, this could be because they used one session of 20-s stretches, while the stretching exercise program in this study was three sessions a week on even days for eight weeks and the stretch was held for 30-s. On the other hand, we used active stretching exercises too, because contraction of the antagonist's muscles might help decrease muscle tightness with the help of reciprocal inhibition.

In the current study, the results show that the significant differences in ODI, VAS, and hip extension PROM between before and after stretching protocol (pre-post) in the experimental group. Therefore, the results of this study can confirm the results of previous studies [21]. Accordingly, pain and disability are the most important factors in preventing success in the treatment of NSLBP. Research has shown that motion therapy improves pain and disability. Several clinical studies have suggested motion therapy to control NSLBP because it has great therapeutic potential for pain relief and disability [22]. Overall, the participants in this study had NSLBP and limited hip extensor ROM. We observed the Positive effect of the static stretch of hip exercise in the study's outcomes. Therefore, these findings suggest that static stretch of hip flexors can be used as an effective exercise program in participants with NSLBP and limited hip extension.

This study has several limitations. First, because the age and sex of the patients were set limited to 25 to 40 years females, our data cannot be generalized to all ages and sex. Second, in our study, the menstrual cycle of the participants in the pre-test and post-test stages was not taken into account and because it is possible for some people to experience NSLBP within their period, it is suggested that the factor be considered in future research. Third, the stretching exercise program was only compared with a no stretching exercise program rather than a placebo condition; thus, Improvement in the evaluated parameters may relate to a placebo effect. Forth, results can be affected by VAS reliability.

## Conclusion

The results demonstrated a significant difference in PROM, pain, and disability after 8 weeks of hip static stretching exercises in participants with NSLBP and limited hip extension. Therefore, it would be reasonable to infer that NSLBP might be partly related to hip flexors tightness.

## Abbreviations

NSLBP: Non-specific low back pain
PROM: Passive hip range of motion
VAS: Visual analog scale
ODI: Oswestry low back pain disability questionnaire

## References

[1] Harris-Hayes M, Sahrmann SA, Van Dillen LR (2009). Relationship between the hip and low back pain in athletes who participate in rotation-related sports. J Sport Rehabil.

[2] Kim WD, Shin DC (2020). Correlations between hip extension range of motion, hip extension asymmetry, and compensatory lumbar movement in patients with nonspecific chronic low back pain. Med Sci Monit.

[3] Roach SM, San Juan JG, Suprak DN, Lyda M, Bies AJ, Boydston CR (2015). Passive hip range of motion is reduced in active subjects with chronic low back pain compared to controls. Int J Sports Phys Ther.

[4] Van Dillen LR, Gombatto SP, Collins DR, Engsberg JR, Sahrmann SA (2007). Symmetry of Timing of Hip and Lumbopelvic Rotation Motion in 2 Different Subgroups of People With Low Back Pain. Arch Phys Med Rehabil.

[5] Mine K (2017). Immediate Effects of Stretching for Iliopsoas Muscles on Lumbopelvic-Hip Kinematics during Gait: A Randomised Controlled Trial Using Subjects with Non-Specific Low Back Pain. Int J Sports Exerc Med.

[6] Thambyah A, Hee HT, De Das S, Lee SM (2003). Gait adaptations in patients with longstanding hip fusion. J Orthop Surg.

[7] Jo HJ, Song AY, Lee KJ, Lee DC, Kim YH, Sung PS (2011). A kinematic analysis of relative stability of the lower extremities between subjects with and without chronic low back pain. Eur Spine J.

[8] Gabbe BJ, Bennell KL, Finch CF (2006). Why are older Australian football players at greater risk of hamstring injury?. J Sports Sci Med.

[9] Vigotsky AD, Lehman GJ, Beardsley C, Contreras B, Chung B, Feser EH (2016) The modified Thomas test is not a valid measure of hip extension unless pelvic tilt is controlled. PeerJ 4:e2325 10.7717/peerj.2325 PMC4991856 27602291

[10] Kolber MJ, Fiebert IM (2005). Addressing flexibility of the rectus femoris in the athlete with low back pain. Strength Cond J.

[11] Winters MV, Blake CG, Trost JS, Marcello-Brinker TB, Lowe L, Garber MB, Wainner RS (2004). Passive versus active stretching of hip flexor muscles in subjects with limited hip extension: A randomized clinical trial. Phys Ther.

[12] Younis Aslan HI, Buddhadev HH, Suprak DN, San Juan JG (2018). Acute Effects of Two Hip Flexor Stretching Techniques on Knee Joint Position Sense and Balance. Int J Sports Phys Ther.

[13] Peterson Kendall F, Kendall McCreary E, Geise Provance P, McIntyre Rodgers M, Anthony Romani W (2005) Muscles Testing and Function with Posture and Pain. 5th ed. Baltimore: Lippincott Williams & Wilkins.

[14] Watt JR, Jackson K, Franz JR, Dicharry J, Evans J, Kerrigan DC (2011). Effect of a supervised hip flexor stretching program on gait in elderly individuals. PM & R.

[15] Matsuo S, Suzuki S, Iwata M, Hatano G, Nosaka K (2015). Changes in force and stiffness after static stretching of eccentrically-damaged hamstrings. Eur J Appl Physiol.

[16] Bae HI, Kim DY, Sung YH (2017). Effects of a static stretch using a load on low back pain patients with shortened tensor fascia lata. J Exerc Rehabil.

[17] Jane Scott P, Huskisson EC (1977). Measurement of functional capacity with visual analogue scales. Rheumatol.

[18] Bartlett MD, Wolff LS, Shurtleff DB, Stahell LT (1985). Hip flexion contractures: A comparison of measurement methods. Arch Phys Med Rehabil.

[19] Roach S, San Juan JG, Suprak DN, Lyda M (2013). Concurrent validity of digital inclinometer and universal goniometer in assessing passive hip mobility in healthy subjects. Int J Sports Phys Ther.

[20] Paatelma M, Karvonen E, Heiskanen J (2009). Clinical perspective: How do clinical test results differentiate chronic and subacute low back pain patients from “non-patients”. J Man Manip Ther.

[21] Minshull C, Eston R, Bailey A, Rees D, Gleeson N (2014). The differential effects of PNF versus passive stretch conditioning on neuromuscular performance. Eur J Sport Sci.

[22] Sung PS (2003). Multifidi muscles median frequency before and after spinal stabilization exercises. Arch Phys Med Rehabil.

